# Beyond Mutation Diversity: Common Pathogenic Mechanism for Putative Intervention Sites in Monogenic Diseases

**DOI:** 10.3390/ijms27219432

**Published:** 2026-10-22

**Authors:** Bikash KC, Nirav Modha, Pitambar Poudel, Shailesh Kumar Panday, Emil Alexov

**Affiliations:** 1 Department of Physics and Astronomy, College of Science, Clemson University, Clemson, SC 29634, USA; bikashk@clemson.edu (B.K.); nmodha@clemson.edu (N.M.); pitambp@g.clemson.edu (P.P.); spanday@clemson.edu (S.K.P.); 2 Medical Biophysics Graduate Program, College of Science, Clemson University, Clemson, SC 29634, USA; 3 Institute for Human Genetics, Clemson University, Greenwood, SC 29646, USA

**Keywords:** proteins, monogenic diseases, molecular dynamics simulations, mutation

## Abstract

Monogenic disorders present a compelling target for drug discovery due to their clear, single-gene etiology. Yet, effective therapies remain scarce, largely because diverse mutation types scattered across a protein obstruct targeted drug design. In this study, we leverage MOGEDO, a curated database of monogenic disorders, to test whether different pathogenic genotypes within a single protein converge on a common molecular mechanism. By clustering MOGEDO proteins into eight functional families and analyzing representative proteins carrying both benign and pathogenic mutations, we integrated molecular dynamics, network analysis, and side-chain entropy metrics. Our findings reveal that distinct pathogenic mutations disrupt the structural and dynamic properties of the wild-type and benign protein through common pathways. By demonstrating that diverse disease-causing mutations manifest a unified pathogenic phenotype, this work provides a framework for designing broad-spectrum therapeutics that target common putative intervention sites rather than individual mutations.

## 1. Introduction

Monogenic diseases are disorders caused by pathogenic variants in a single gene [1], and although individual conditions are usually rare, they collectively create a substantial health burden. Rare diseases as a group affect an estimated 3.5–5.9% of the global population, and approximately 80% of recognized rare diseases are thought to result from defects in a single gene [1,2]. A pathogenic variant can reduce protein function, alter protein stability, disrupt molecular interactions, or change normal cellular regulation [3]. Despite progress in molecular diagnosis and genetic medicine, most monogenic and rare diseases still lack an approved treatment that directly eliminates the underlying disease mechanism. Consequently, clinical care often focuses on symptom control, supportive care, or management of complications [4].

Developing targeted therapies for monogenic diseases presents a unique set of challenges despite the clear identification of a single causal gene. One reason is that many monogenic disorders are exceptionally rare; consequently, recruiting sufficient participants for clinical trials is exceptionally difficult, often stalling trials or limiting statistical power [5]. Furthermore, knowing the mutated gene does not always clarify how the protein alteration disrupts physiological pathways, making it hard to design molecules that effectively restore normal function. In addition, many single-gene conditions affect early embryonic development or cause progressive, irreversible neurodegeneration before clinical symptoms appear, rendering late-stage pharmacological intervention ineffective [6,7]. Delivery presents an additional challenge: targeting intracellular or central nervous system pathways, including crossing the blood–brain barrier, often requires specialized delivery systems that carry risks of immune rejection, off-target toxicity, and high production complexity [7,8].

A single monogenic disease gene can contain many different pathogenic variants, referred to as different genotypes. These variants may include missense substitutions, nonsense mutations, insertions or deletions, and splice-site changes [3,9]. Even when they occur in the same gene, they may not necessarily affect the encoded protein in the same way. Some variants may alter protein structure, whereas others may alter catalytic activity, change conformational flexibility, disrupt protein–protein interactions, or affect cellular localization and signaling [3,10]. Therefore, different mutations in the same gene may affect the protein’s function by different molecular mechanisms [11]. Thus, different mutations in the same gene, along with the genetic background of the individual, could cause some patients to develop more severe symptoms, show symptoms earlier, or have different clinical features than others. Perhaps this is one of the factors impairing drug development, along with the common expectation that a treatment that works for one mutation may not work well for another pathogenic mutation in the same protein [12].

Identifying the disease-causing gene does not automatically lead to an effective treatment [13]. For many monogenic disorders, current care is still directed toward reducing symptoms, preventing complications, and improving quality of life rather than correcting the underlying molecular defect [4,14]. Disease-specific medicines, enzyme-replacement therapies, RNA therapies, and gene therapies have improved outcomes for some conditions. However, these options are not available for most monogenic diseases, and their benefit can depend on the affected tissue, the type of mutation, and the ability to deliver treatment safely and durably [15]. Thus, there remains a need for therapeutic approaches that act on shared disease-related protein defects and can potentially benefit patients carrying different pathogenic variants in the same gene [16].

Traditional small-molecule drug discovery has often focused on the active or natural ligand-binding site of a protein [17]. Drugs designed for these sites commonly act as competitive inhibitors by blocking substrate or ligand binding [18]. This approach has been highly successful for many enzymes and receptors. However, it will not be suitable when pathogenic variants already reduce protein activity, because further inhibition could worsen the functional defect [19,20]. In such cases, a therapeutic strategy should be to design drugs that selectively modulate protein function [21]. Allosteric sites and small molecules bound there provide one possible way to achieve this type of regulation [22]. However, identifying such allosteric sites requires complex analysis of the disease-causing effect and how it can be mitigated by a small molecule bound at the allosteric site [23].

Developing one treatment for many disease-causing mutations in the same protein requires identifying a molecular defect that is shared across those mutations [24]. The mutations may occur at different positions in the sequence, but they can still converge on a common outcome, such as reduced protein stability, altered conformational dynamics, or disruption of the same regulatory pathway [25,26]. If this shared defect can be identified, a drug could be designed to stabilize or modulate the affected protein or pathway and may benefit patients with several, rather than only one, pathogenic variant [27]. This approach is not expected to work for every mutation, because some variants may act through distinct mechanisms. However, identifying groups of variants with a common molecular phenotype could make therapeutic development more efficient and expand the number of patients who may benefit from a single treatment [28].

Previous studies support the concept that pathogenic variants at different positions can converge on a shared molecular phenotype. For example, molecular-dynamics analysis of ten familial prion-protein variants found common alterations in protein dynamics and intermolecular-recognition regions, despite preservation of the overall native fold. These changes converged on remodeling of the β2-α2 loop, which was proposed as a common therapeutic-interference target [29]. More broadly, protein structural-network analyses [30] have shown that disease-causing mutations can produce long-range changes in residue-interaction networks, including at functionally important sites distant from the mutation itself [31]. Recent works on arginine-to-histidine variants [32] and variants involving charged groups [33] demonstrated that such mutations affect pH-dependent properties of the corresponding proteins. Together, these findings suggest that distinct pathogenic variants may converge on shared structural, dynamic, or network-level defects that could be exploited for therapeutic development.

The goal of this study was to determine whether different missense pathogenic variants in the same monogenic disease protein cause shared structural and dynamical changes. While there are many works, mentioned above, that seek to reveal molecular mechanisms of various mutations within the same protein, our investigation goes one step further to identify common putative intervention sites. We selected eight representative proteins from the MOGEDO database, which contain curated missense pathogenic and benign variants associated with monogenic disorders [34]. For each protein, we compared the wild-type system, a benign variant, and multiple pathogenic variants using molecular-dynamics simulations. We then combined structural stability measures, residue interaction-network analysis, and side-chain conformational entropy analysis to identify residues and communication pathways that are consistently altered by pathogenic variants. Rather than predicting a drug directly, this work aims to identify shared molecular defects that can help prioritize common therapeutic targets for multiple pathogenic variants in the same protein.

## 2. Results

### 2.1. Selection of Functionally Diverse Protein Targets

The initial protein set was derived from the MOGEDO pathogenic protein dataset [34]. The MOGEDO dataset used in this study contained 3339 pathogenic and benign variant records spanning over 686 genes and 700 RefSeq protein accessions. To allow for structure-based analysis, these records were first consolidated into a protein-centered dataset, from which 309 proteins with validated structural mappings were identified. The structural dataset was then screened for experimentally resolved wild-type structures suitable for further downstream mutation-focused analysis. A restriction to strict wild-type X-ray structures yielded 204 proteins, all represented by 1733 PDB structural candidates. Further filtering based on sequence and structural coverage of the relevant pathogenic and benign mutation positions reduced the set to 870 candidate structures representing 121 proteins. The progression of protein and structural candidates through the screening stages is summarized in Supplementary Table S3A. The 121 proteins retained after structural and mutation-site coverage filtering, along with their selection status for molecular dynamics (MD) simulations, are detailed in Supplementary Table S3B, while the 83 excluded proteins are listed in Supplementary Table S3C. The distribution of proteins across functional subclasses and the final selection of eight representative protein targets for downstream MD simulations are presented in Supplementary Table S3D.

Running three replicates of one-microsecond-scale molecular dynamics simulations for every structurally eligible wild-type protein and its associated benign and pathogenic variants would have been quite computationally expensive and technically prohibitive. Therefore, we used a representative strategy that retains functional diversity while also prioritizing proteins with suitable structural similarity and mutation coverage. Structure- and function-based screening ultimately reduced the list to eight protein targets representing eight distinct functional subclasses for downstream analysis. The final set consisted of N-acetylgalactosamine-6-sulfatase (GALNS), serine hydroxymethyltransferase, mitochondrial (SHMT2), dihydropyrimidinase (DPYS), Nectin-4 (NECTIN4), death domain-containing protein CRADD (CRADD), EKC/KEOPS complex subunit TP53RK (TP53RK), glycosylphosphatidylinositol-anchored high-density lipoprotein-binding protein 1 (GPIHBP1), and fructose-1,6-bisphosphatase 1 (FBP1), representing the other enzyme, DNA/RNA-binding, binding, receptor, regulatory, kinase, transporter, and phosphatase subclasses, respectively (Table 1). Representative experimental structures were then selected for each protein based on structural representativeness, mutation-position coverage, and suitability for downstream structural preparation (see Section 4 for details).

For each of these eight proteins, the wild-type, pathogenic, and benign systems were subjected to three independent MD simulation runs (three replicas), each 1 μs long; the specific benign and pathogenic variants analyzed for each protein are listed in Supplementary Table S4. The resulting trajectories were then used to carry out network and side-chain entropy analyses to identify pathogenic-mutation-induced perturbations in the wild-type and benign-variant protein systems.

### 2.2. Network Analysis

The central goal of the network analysis was to determine whether pathogenic mutations occurring at different positions within the same protein converge on a common disruption of the protein’s internal communication network. To establish a nonpathogenic baseline, we first identified network features that were preserved between the wild-type (WT) protein and its benign variant. These shared features between WT and benign were used to define a protein-specific WT/benign reference signature representing the functional protein network, termed the network reference signature (NRS). It should be clarified that no attempt was made to find the functional validation of the NRS, and it is possible that it may not be in a functionally preserved state. Then, each pathogenic variant was compared independently with the NRS to see which network features were consistently altered. We reasoned that network perturbations recurring across multiple pathogenic variants, despite the mutations occurring at different positions, would provide evidence for a shared pathogenic mechanism.

Network analysis (detailed in Section 4.6) was therefore performed for the WT, benign, and pathogenic systems of each of the eight representative proteins. In a residue-interaction network, residues act as nodes whose connections reflect coordinated structural interactions and communication through the protein. The WT and benign systems were first used to construct the WT/benign reference signature described above. Pathogenic systems were then compared with this signature, the NRS, to identify Network Gain and Loss residues. A Gain represents a highly ranked network position appearing in the pathogenic system but not in the NRS, whereas a Loss represents a reference network position in the NRS that is no longer preserved in the pathogenic system. Recurrent Gain and Loss events were observed in all eight proteins, although their number and frequency varied among the systems. These recurrent changes identify positions whose network topology is repeatedly altered by pathogenic mutations and therefore represent candidate sites of shared network-level perturbation.

**Figure 1:**
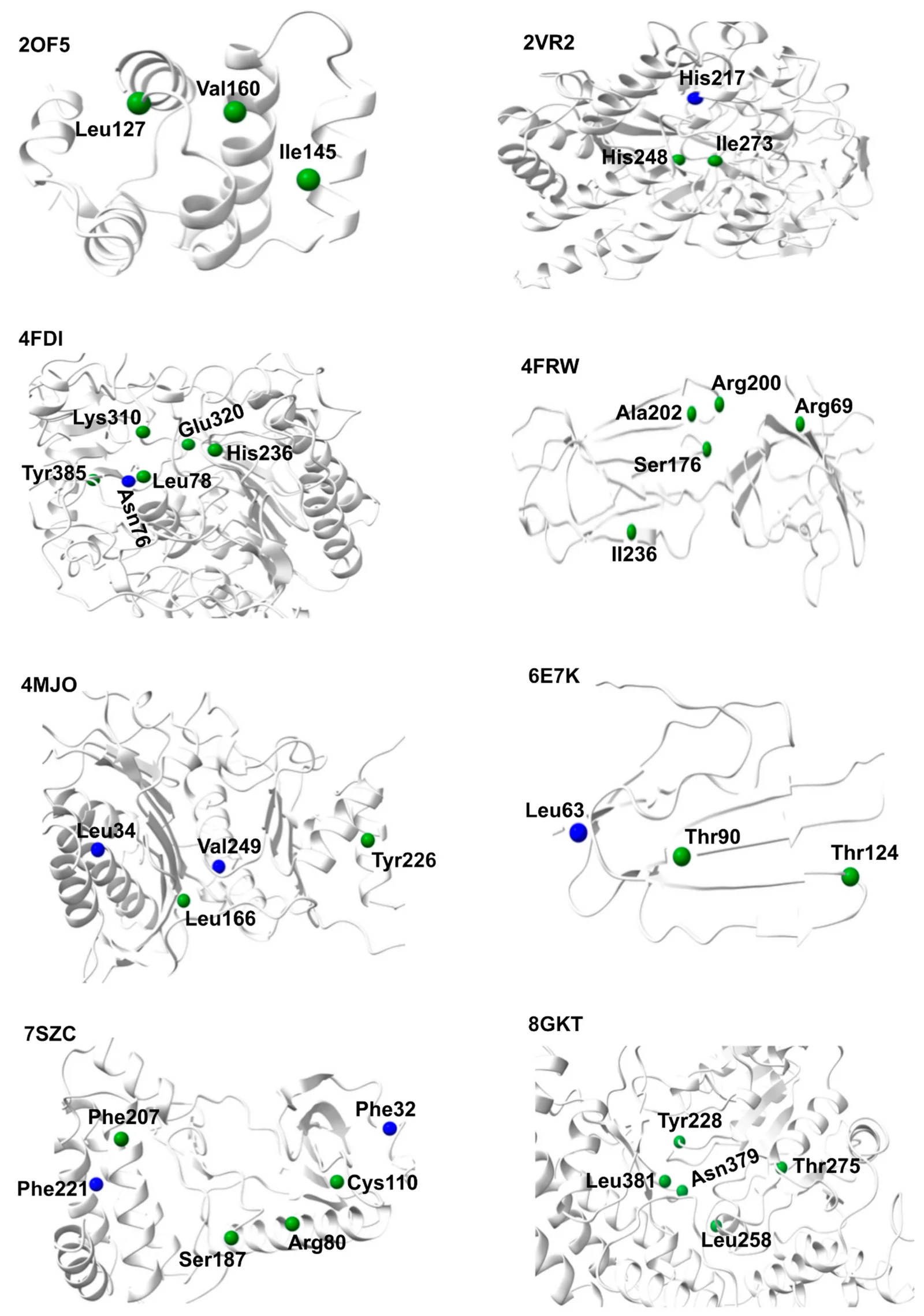
Structural localization of recurrent Network Gain and Network Loss residues across the eight investigated proteins. Recurrent network residues identified across the pathogenic variants are mapped onto the representative structures of 2OF5, 2VR2, 4FDI, 4FRW, 4MJO, 6E7K, 7SZC, and 8GKT. Network Gain residues are shown in green and Network Loss residues in blue. Network Gain refers to a pathogenic top-25 bottleneck residue without direct or spatial correspondence to the WT/benign Network Reference Signature (NRS), whereas Network Loss refers to an NRS residue without direct or spatial correspondence to the pathogenic top-25 bottleneck residues. Spatial correspondence was evaluated using a 5.0 Å Cα–Cα distance threshold. Residue labels follow the original RCSB PDB numbering, which corresponds to UniProt numbering except for 4MJO, where the UniProt residue number equals the original PDB residue number plus one. Chain A was analyzed for all structures except 6E7K, for which chain C was selected. The structures represent MD-derived average structures calculated from the 1-μs production trajectories and were visualized using ChimeraX (version 1.12).

The recurrent Network Gain and Loss residues identified across the eight proteins and the corresponding mutants are summarized in Table 2. To determine the spatial localization of these recurrent network perturbations, the corresponding residues were also mapped onto each representative PDB structure (Figure 1). Recurrent network alterations were observed across all eight systems, showing that pathogenic mutations can repeatedly affect common positions within the NRS within the same protein.

The detailed comparison of the NRS with each pathogenic variant is provided in Supplementary Table S5A, which summarizes the reference residues classified as Shared Direct, Shared Spatial, or Network Loss. The complete set of pathogenic Network Gain residues and their bottleneck scores is provided in Supplementary Table S5B. Following these comparisons, most NRS positions were preserved across the pathogenic variants, although the extent of preservation varied among the eight proteins.

To provide more specific details, here we outline the results obtained. Thus, for CRADD (PDB ID: 2OF5) protein, 24 of the 25 NRS positions were shared in each of the three pathogenic variants. In DPYS (PDB ID: 2VR2) protein, all 19 reference positions were shared in R412M mutant, while 17 were shared in G435R and R490C mutants, 16 in V59F mutant, and 14 in M250I and Q334R mutants. In GALNS (PDB ID: 4FDI) protein, 18 of the 19 reference positions were shared in R90W, S162F, and S287L mutants, 17 in M391V mutant, and 15 in G139S and M318R mutants. For NECTIN4 (PDB ID: 4FRW) protein, all 20 reference positions were shared in P212R and V242M mutants, whereas 19 were shared in T185M mutant. Furthermore, in FBP1 (PDB ID: 4MJO) protein, all 19 NRS positions were shared in G260R mutant, while 17 were shared in A177D mutant, 16 in F194S, P284R, and R158W mutants, and 15 in G164S mutant. In GPIHBP1 (PDB ID: 6E7K) protein, 23 of the 24 NRS positions were shared in C65Y, C68G, and T111P mutants, whereas 22 were shared in C89F, Q115P, and S107C mutants. In TP53RK (PDB ID: 7SZC) protein, 19 of the 22 NRS positions were shared in G42D mutant, while 20 were shared in R243L and T81R mutants. Finally, in SHMT2 (PDB ID: 8GKT) protein, all 20 reference positions were shared in P157S mutant, 17 were shared in P499A mutant, and 16 were shared in G423S and Q435P mutants.

Although most reference positions were preserved, each pathogenic system also showed Gain and/or Loss of bottleneck positions. Because the major goal of this study was to identify network changes shared by different pathogenic variants, we next focused on Gain and Loss positions that recurred across multiple pathogenic variants. For CRADD, the Gain position I145 is identified in all three pathogenic variants, while the Gain positions L127 and V160 are identified in two of the three variants. In the DPYS system, the Gain position H248 is identified in five of the six pathogenic variants and the Gain position I273 in four variants, whereas position H217 is shown as Network Loss in four variants. Furthermore, for GALNS, the Gain position K310 is identified in all six pathogenic variants, while positions H236 and Y385 are identified in five variants, positions L78 and E320 in four variants, and finally the position N76 is shown as Loss in five variants. Analysis of NECTIN4 showed that positions R200, A202, and I236 are identified as Network Gain in all three pathogenic variants, while positions R69 and S176 are identified in two variants. For FBP1, position Y226 is found as a Gain in all six pathogenic variants and position L166 in four variants, whereas position V249 is marked as a Loss in five variants and position L34 in four variants. In the GPIHBP1 system, position T90 is indicated as a Gain in five of the six pathogenic variants and position T124 in three variants, whereas position L63 is found to be Loss in three variants. For TP53RK, positions R80 and F207 were marked as a Gain in all three pathogenic variants, while positions C110 and S187 were identified as a Gain in two variants; positions F32 and F221 were identified as a Loss in two variants. Analysis of SHMT2 revealed that positions Y228, L258, T275, N379, and L381 were marked as a Network Gain in three of the four pathogenic variants.

Together, these results show that although much of the WT/benign reference network remained preserved, pathogenic variants repeatedly introduced Gain and Loss events at specific bottleneck positions. These recurrent changes identify protein-specific network perturbations shared across different pathogenic variants and were therefore carried forward for comparison with the side-chain entropy results and structural analysis.

### 2.3. Side-Chain Entropy Analysis

In addition to the long-range communication changes identified by network analysis, pathogenic mutations may also alter the local conformational behavior of individual amino acid side-chains. Therefore, side-chain entropy analysis was performed as a complementary approach to characterize residue-level changes in side-chain conformational sampling and to determine whether different pathogenic mutations repeatedly perturb the same amino acid residues. Side-chain entropy reflects how broadly a residue occupies different side-chain conformations during the simulation; a change in entropy relative to WT therefore shows altered local conformational behavior.

Like the network analysis, the benign variant was used as a nonpathogenic reference. Residues showing an absolute normalized entropy change above the selected threshold (|*ΔS*| > 0.10) (|*ΔS*| indicates the absolute normalized entropy) in the pathogenic systems, while remaining comparatively unchanged in the benign system (|*ΔS*| < 0.05), were considered as perturbed. Recurrently perturbed residues identified across the eight proteins are summarized in Table 3, while the complete |*ΔS*| values for the benign and pathogenic variants are provided in Supplementary Table S2.

For CRADD, position L194 was perturbed in two of the three pathogenic variants, with |*ΔS*| values ranging from 0.144 to 0.154. For DPYS, position W120 showed the highest recurrence and was perturbed in all six pathogenic variants, with |*ΔS*| values ranging from 0.137 to 0.242. Positions Q71, H69, and R221 were each perturbed in five of the six variants, with |*ΔS*| ranges of 0.123–0.327, 0.131–0.403, and 0.155–0.227, respectively, whereas position N347 was perturbed in four variants, with values ranging from 0.296 to 0.400. For GALNS, positions R253, R105, Y108, and H398 were each perturbed in five of the six pathogenic variants, with |*ΔS*| ranges of 0.210–0.321, 0.113–0.272, 0.164–0.258, and 0.110–0.196, respectively. Positions N289, L395, and F167 were perturbed in four of the six variants, with |*ΔS*| ranges of 0.319–0.409, 0.146–0.262, and 0.143–0.249, respectively. Analysis of NECTIN4 showed that positions M216, Q163, and R214 were perturbed in all three pathogenic variants, with |*ΔS*| ranges of 0.210–0.341, 0.165–0.362, and 0.108–0.204, respectively, while position R97 was perturbed in two of the three variants, with values ranging from 0.143 to 0.275. For FBP1, positions N212 and D323 showed the highest recurrence and were perturbed in all six pathogenic variants, with |*ΔS*| ranges of 0.170–0.279 and 0.101–0.193, respectively. Positions F117 and K230 were each perturbed in five of the six variants, with |*ΔS*| ranges of 0.200–0.345 and 0.124–0.169, respectively, whereas positions P233 and D251 were perturbed in four variants, with ranges of 0.102–0.125 and 0.162–0.266, respectively. In the GPIHBP1 system, positions L63 and R64 were each perturbed in two of the six pathogenic variants, with |*ΔS*| ranges of 0.170–0.195 and 0.108–0.159, respectively. Position E122 was also perturbed in two variants, with values ranging from 0.114 to 0.131. For TP53RK, positions N109 and Y106 were each perturbed in two of the three pathogenic variants, with |*ΔS*| ranges of 0.135–0.262 and 0.109–0.283, respectively. For SHMT2, positions Y106 and H171 showed the highest recurrence and were each perturbed in three of the four pathogenic variants, with |*ΔS*| ranges of 0.120–0.184 and 0.111–0.219, respectively, whereas positions Y141, M498, and Q324 were each perturbed in two of the four variants, with |*ΔS*| ranges of 0.179–0.404, 0.222–0.223, and 0.217–0.229, respectively.

Together, these results show that pathogenic mutations at different positions can commonly perturb the conformational behavior of the same residues within an individual protein. Although the magnitude of the entropy change varied among pathogenic variants, the recurrence of these perturbations identifies candidate local conformational signatures shared across multiple pathogenic mutations. These residues were subsequently integrated with the recurrent network perturbations and their spatial relationships to identify common candidate patches.

### 2.4. Unified Analysis and Identification of Candidate Patches

**Figure 2:**
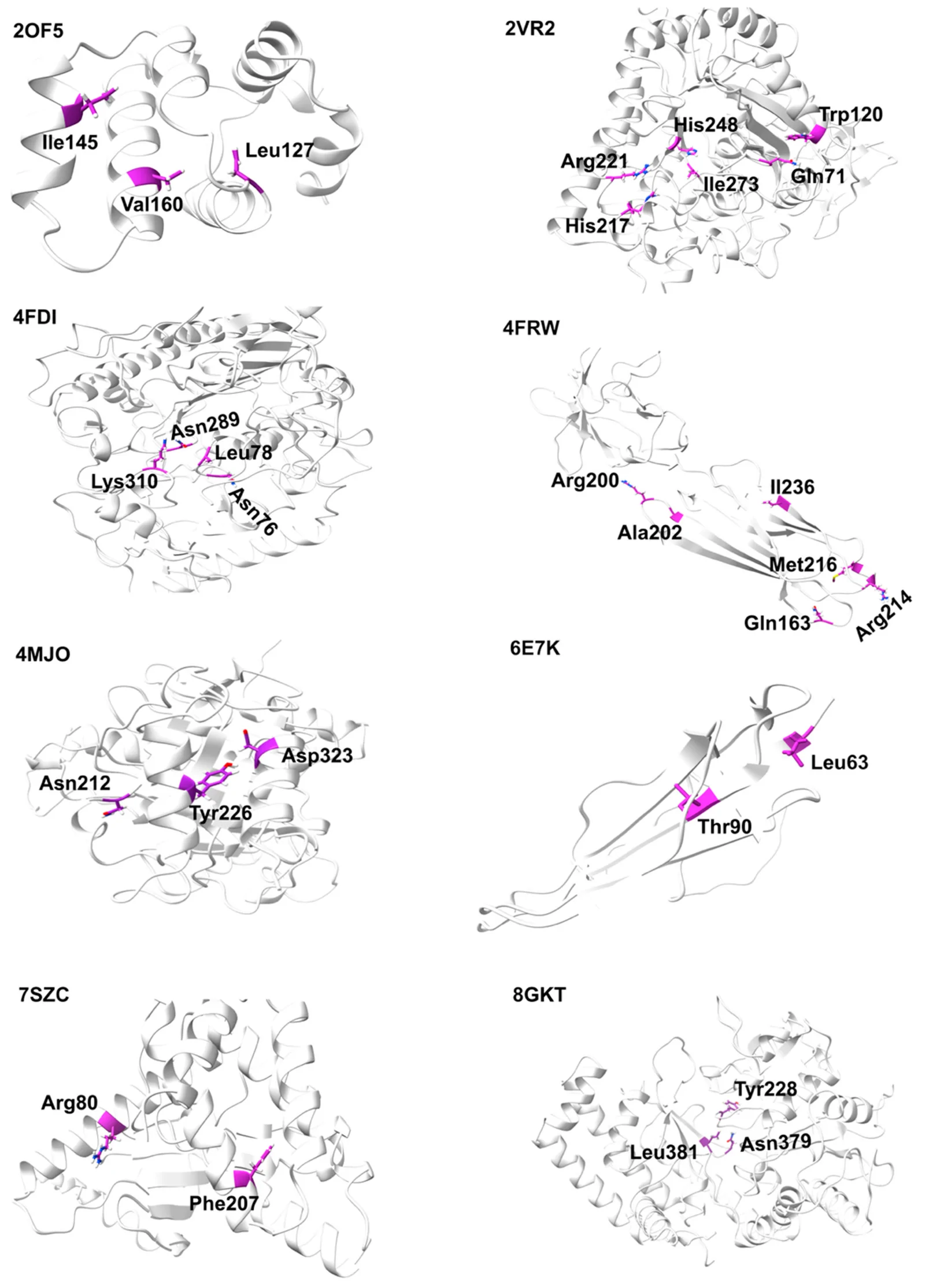
Cartoon representation of the candidate patches identified by the unified analysis across the eight investigated proteins. Residues defining the candidate patches are shown in magenta and labeled on the representative structures of PDB: 2OF5, 2VR2, 4FDI, 4FRW, 4MJO, 6E7K, 7SZC, and 8GKT. Residue labels follow the original RCSB PDB numbering, which corresponds to UniProt numbering except for 4MJO, where the UniProt residue number equals the original PDB residue number plus one. Chain A was used for all protein structures except 6E7K, for which chain C was selected. Structures were visualized using ChimeraX (version 1.12).

**Figure 3:**
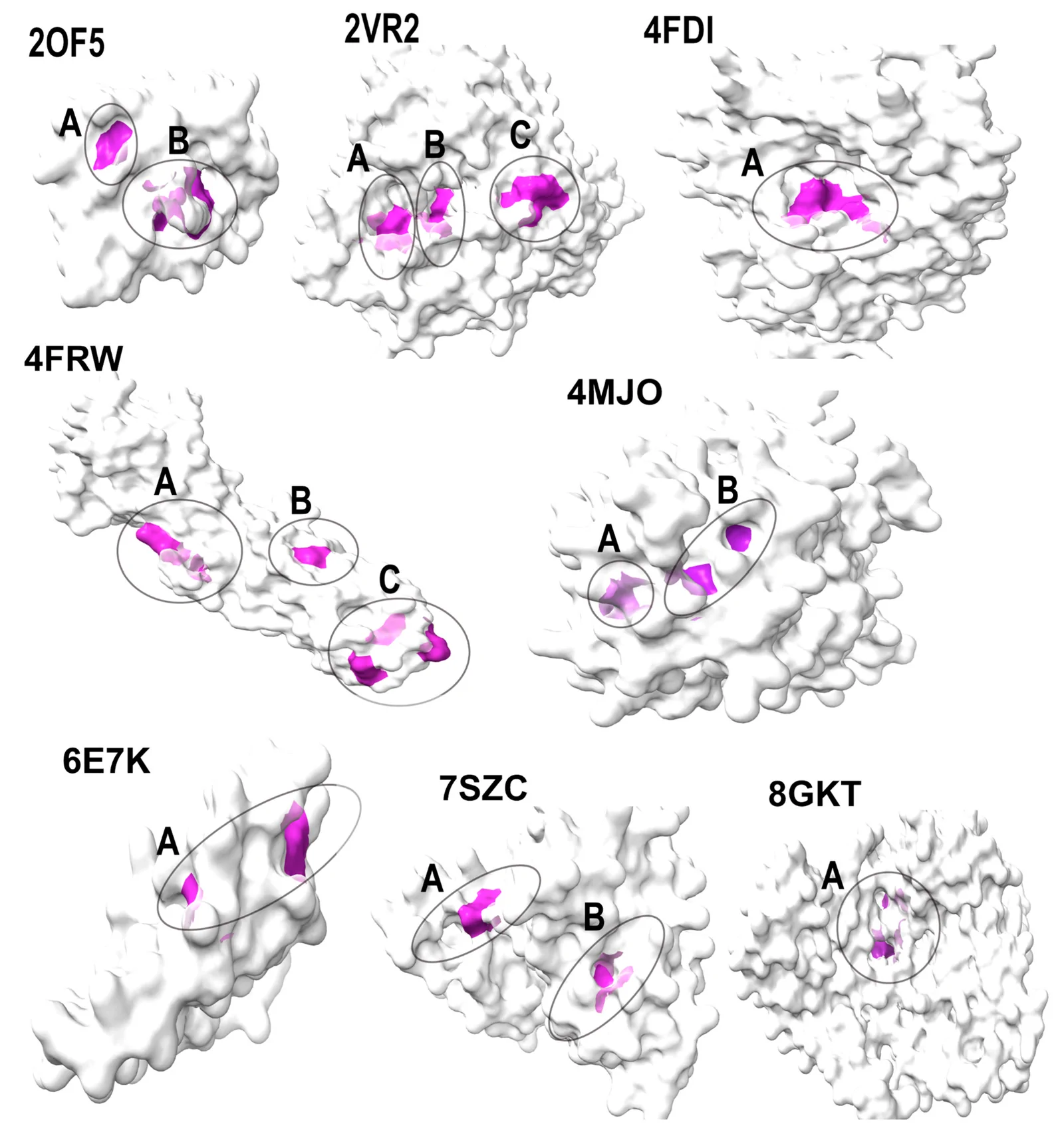
Surface representation of the candidate patches identified by the unified analysis across the eight representative proteins. The same candidate patches shown in Figure 2 are displayed as magenta surface regions on the representative structures of 2OF5, 2VR2, 4FDI, 4FRW, 4MJO, 6E7K, 7SZC, and 8GKT. Labels A, B, and C indicate distinct candidate patches within each protein and correspond to the patches listed in Supplementary Table S1. Residue numbering follows the original PDB coordinates (4MJO: UniProt = PDB + 1), with chain A used except for 6E7K (chain C). Structures were visualized using ChimeraX (version 1.12).

The network and side-chain entropy analyses captured different aspects of pathogenic perturbation. Network analysis identified common changes in residue communication, whereas side-chain entropy identified common changes in local conformational behavior. The recurrent residues identified by the two analyses were then integrated with their spatial relationships to define candidate patches for each protein. A candidate patch could be supported by network analysis, side-chain entropy analysis, or both when the identified residues occupied the same local structural region. Residues present in at least half of the pathogenic variants were considered, and residues with different recurrence patterns were grouped when they occupied the same local structural region and collectively represented the pathogenic variants. Residues identified in all pathogenic variants were either grouped with nearby selected residues or retained as individual candidate patches when spatially separated. The resulting regions were further examined in relation to previously reported functionally or structurally important residues. The candidate patches identified across the eight proteins are mapped onto their representative structures in Figure 2 and summarized in Figure 3.

Based on the network results described above for CRADD protein, positions I145, L127, and V160 were carried forward for the unified analysis. Although positions L127 and V160 were not present in every pathogenic variant, their occurrence was complementary, and together they were represented in all three pathogenic variants. The two residues are separated by 8.91 Å, placing them within the same local structural region. Position I145, in contrast, was retained as an individual site because it was identified in all three pathogenic variants. At this point, it should also be noted that this region is structurally relevant: L127 forms part of the F164-centered hydrophobic cluster together with V135 and L180, which has been associated with the stability of the RAIDD death domain [35]. In addition, V160 lies close to F164, while I145 lies close to V135. These observations support the definition of two candidate patches in CRADD: I145 and the L127–V160 pair.

Similarly, for DPYS protein, based on the network and side-chain entropy results described above, positions W120, Q71, H217, R221, H248, and I273 were carried forward for the unified analysis. Position W120 showed side-chain entropy perturbation in all six pathogenic variants, while position Q71 was perturbed in five variants, and the two residues are located close to each other (8.51 Å). Position H248 showed Network Gain in five of the six variants and was absent only in Q334R, whereas position I273 showed Gain in Q334R along with three other variants. Thus, positions H248 and I273 together cover all six pathogenic variants and are separated by 5.14 Å. Similarly, position H217 showed Network Loss across four variants, whereas position R221 showed side-chain entropy perturbation across five variants; together, they represented all six pathogenic variants. Positions H217 and R221 are separated by 6.74 Å, indicating that the two residues are also located within the same local structural region. These regions are also located close to residues involved in the catalytic site of human dihydropyrimidinase. H248 itself is one of the residues involved in the coordination of the binuclear zinc center, while D326 functions as a catalytic base and zinc ligand, and N347 contributes to substrate binding [36,37]. In our structural analysis, I273 was located 4.06 Å from D326, whereas H217 was 4.93 Å from N347. These relationships place the residues identified by the network and entropy analyses near the catalytic and substrate-binding regions of DPYS. Based on their occurrence across the pathogenic variants and their spatial relationships, three candidate patches were defined in DPYS: Patch A, H217–R221; Patch B, H248–I273; and Patch C, W120–Q71.

Likewise, for GALNS, positions K310, L78, N76, and N289 were carried forward for the unified analysis. K310 showed Network Gain in all six pathogenic variants, while L78 showed Gain in four variants, N76 showed Network Loss in five variants, and N289 showed side-chain entropy perturbation in four variants. These four residues are also located close to one another, with pairwise distances ranging from 5.56 to 9.62 Å, indicating that the different network and entropy changes occur within the same local structural region. K310, therefore, represents the residue common to all six pathogenic variants, while L78, N76, and N289 define the surrounding region affected in multiple variants. This region also closely corresponds to the active site of GALNS. Previous structural studies identified K310 and N289 as active-site residues of GALNS. N289 also helps coordinate the catalytic Ca^2+^ ion, while residue 79, located next to L78, serves as the catalytic nucleophile [38]. Thus, the residues selected from the network and entropy analyses are concentrated around an established catalytic region of GALNS. Based on their occurrence across the pathogenic variants and their spatial relationships, K310–L78–N76–N289 was defined as a candidate patch in GALNS protein.

For NECTIN4, based on the network and side-chain entropy results described above, residues R200, A202, I236, M216, Q163, and R214 were carried forward for the unified analysis. R200, A202, and I236 showed Network Gain in all three pathogenic variants, while M216, Q163, and R214 showed side-chain entropy perturbation in all three variants. R200 and A202 were located close to each other, with a Cα–Cα distance of 6.24 Å, and were therefore grouped within the same local structural region. M216 and R214 also formed a close pair, with a Cα–Cα distance of 5.28 Å. Although Q163 was farther from these residues based on Cα–Cα distance, its side-chain approached that of M216 within 9.88 Å, supporting a spatial connection between Q163 and the M216–R214 pair. Q163, M216, and R214 were therefore grouped as a single connected candidate patch. In contrast, I236 was spatially separated from the other selected residues and was retained as an individual candidate patch. These residues are located outside the well-characterized D1 adhesive interface of Nectin-4. Previous structural studies showed that the canonical adhesive interface is formed mainly by the BC, C′C″, and FG loops of the D1 domain, which are involved in Nectin-4 interactions with its cellular and viral binding partners [39]. The regions identified here are instead located in the more membrane-proximal part of the protein, suggesting that the changes observed in the network and entropy analyses occur at sites distinct from the established D1 interaction surface. Based on their occurrence across the pathogenic variants and their spatial relationships, three candidate patches were defined in NECTIN4: Patch A, R200–A202; Patch B, I236; and Patch C, Q163–M216–R214.

Turning to FBP1 protein, residues Y226, N212, and D323 were carried forward for the unified analysis. Y226 showed Network Gain in all six pathogenic variants, while N212 and D323 showed side-chain entropy perturbation in all six variants. Y226 and D323 are located close to each other (9.91 Å) and were therefore grouped within the same local structural region. In contrast, N212 is spatially separated from this pair and was retained as an individual candidate site. Additionally, we noted that N212 has previously been associated with the substrate-binding region of fructose-1,6-bisphosphatase. Structural studies identified Asn212 as one of the residues contributing to the fructose-2,6-bisphosphate binding site, together with several neighboring residues [40]. This provides additional functional support for N212, while the Y226–D323 pair represents a second region identified from our network, entropy, and spatial analyses. Based on their occurrence across the pathogenic variants and their spatial relationships, two candidate patches were defined in FBP1: Patch A, N212 and Patch B, Y226–D323.

For GPIHBP1, based on the network and side-chain entropy results described above, T90 and L63 were carried forward for the unified analysis. T90 showed Network Gain in five of the six pathogenic variants, whereas L63 showed Network Loss in three variants. Importantly, T90 was not identified in C65Y, while L63 showed Network Loss in this variant. Thus, although neither residue was present in all six pathogenic variants individually, together L63 and T90 represented all six pathogenic variants. L63 and T90 have a Cα–Cα distance of 11.91 Å, while their side-chains approach within approximately 9.93 Å, indicating that the two residues occupy neighboring regions of the protein structure. Based on their collective representation across the pathogenic variants and their spatial relationship, L63–T90 was defined as a candidate patch in GPIHBP1.

Similarly, for TP53RK protein, based on the network and side-chain entropy results described above, R80 and F207 were carried forward for the unified analysis. Both residues showed Network Gain in all three pathogenic variants, indicating that each residue represents a common network change across the pathogenic systems. We further noted that both residues are located close to structurally important regions of PRPK. R80 is located close to E84, a conserved kinase residue that forms a salt bridge with K60 and helps maintain the active kinase conformation. F207 itself forms part of the catalytic spine of PRPK, together with residues including L203, and therefore contributes directly to the structural organization of the active kinase [41]. These structural relationships provide additional support for the importance of the two regions identified by the network analysis. Based on their occurrence in all three pathogenic variants and their spatial separation, two individual candidate patches were defined in TP53RK: R80 and F207.

Finally, for SHMT2 protein, based on the network and side-chain entropy results described above, Y228, N379, and L381 were carried forward for the unified analysis. Each residue showed Network Gain in three of the four pathogenic variants. Y228 showed Gain in P157S, P499A, and Q435P, whereas N379 and L381 showed Gain in G423S, P157S, and P499A. Thus, although none of the three residues was identified in all four pathogenic variants individually, together they represented all four pathogenic variants. The three residues are also located close to one another, with pairwise Cα–Cα distances of 8.09 Å for Y228–N379, 6.82 Å for Y228–L381, and 5.73 Å for N379–L381, indicating that they occupy the same local structural region. In addition, N379 has previously been linked to SHMT2 function and disease. A pathogenic N379D variant has been reported in patients with an SHMT2-associated neurodevelopmental disorder [42], and more recent structural studies [43] indicate that N379 participates in a local interaction network near the active-site region of SHMT2. This provides additional support for the functional importance of the region identified in our analysis. Based on their complementary occurrence across the pathogenic variants and their spatial relationships, Y228–N379–L381 was defined as a single candidate patch in SHMT2.

Taken together, the unified analysis identified one or more candidate patches in each of the eight proteins. These patches arose from network perturbations, side-chain entropy changes, or both, and were further supported by their spatial clustering within the protein structures. In several cases, the identified regions were located near previously characterized catalytic, binding, or structurally important residues. Thus, despite the pathogenic mutations occurring at different positions, their effects repeatedly converged on a set of shared structural regions within each protein. These candidate patches provide the basis for considering whether common structural and dynamic perturbations may represent shared mechanistic targets across different pathogenic variants.

## 3. Discussion

The traditional paradigm of targeted drug discovery in monogenic disorders relies heavily on mutation-specific targeting or direct orthosteric modulation. While effective in specific cases, this approach faces intrinsic scalability limits given the massive mutational heterogeneity characteristic of human disease genes. Across thousands of monogenic conditions, hundreds of distinct, non-synonymous variants distributed throughout a single protein sequence can lead to loss of function, altered dynamics, or pathological misfolding. Designing individualized small molecules for every disease-causing allele is economically and logistically unviable. In this study, we addressed a fundamental biophysical question: do disparate pathogenic mutations scattered across a protein’s primary sequence converge on shared, higher-order structural and dynamic perturbations?

By integrating microsecond-scale molecular dynamics (MD) simulations, residue interaction network analysis, and side-chain conformational entropy metrics across eight functionally diverse protein families from the MOGEDO database, our findings demonstrate that distinct pathogenic genotypes do indeed converge onto unified, localized structural mechanisms. These shared perturbation sites termed “candidate shared structural-dynamic perturbation patches” offer a pathway for further investigations to seek therapeutic solutions for broad patient cohorts regardless of their specific underlying mutation.

A central finding of this work is that disease-causing mutations, even those located tens of Angstroms apart, repeatedly trigger network-level reorganizations at the same structural nodes. By comparing pathogenic-variant networks with the curated WT/benign reference network (NRS), we consistently observed recurrent “Gain” and “Loss” bottleneck residues in the pathogenic variants. In dynamic network theory, bottleneck nodes represent structural hubs that mediate information flow, long-range force transmission, and conformational coupling across a protein fold. When diverse pathogenic mutations perturb these hubs, they disrupt long-range allosteric communication. For instance, in galactosamine (N-acetyl)-6-sulfatase (GALNS/PDB: 4FDI), the K310 node exhibited a Network Gain across all six distinct pathogenic variants analyzed (6/6 recurrence), accompanied by recurrent gains and losses at nearby residues L78, N76, and N289. Remarkably, although these mutations span distinct regions of the tertiary structure, they all induce a concentrated reorganization of the residue interaction network centered around the catalytic pocket and its coordinating Ca^2+^ ion. This network convergence indicates that protein structures possess intrinsic, evolutionarily conserved signal transduction pathways. Pathogenic variants disrupt these pathways at sensitive, high-leverage nodes. Rather than causing generalized, non-specific collapse, diverse pathogenic mutations destabilize or rewire specific, highly sensitive communication pathways.

While network analysis captures long-range and backbone-mediated communication pathways, side-chain conformational entropy analysis provides a complementary view of local thermodynamic fluctuations. Our metrics revealed that pathogenic variants induce localized side-chain flexibility changes (∣*ΔS*∣ > 0.10, see Section 4) at specific sites that remain structurally rigid and thermodynamically stable in both wild-type and benign proteins (∣*ΔS*∣ < 0.05). For example, in human dihydropyrimidinase (DPYS/PDB: 2VR2), residue W120 exhibited significant side-chain entropy alterations across all six pathogenic variants (6/6 recurrence), while adjacent residues Q71, H69, and R221 were perturbed in five out of six variants. This local entropy change reflects a heightened sampling of non-native rotameric states, signaling a loss of local packing integrity that propagates toward the binuclear zinc catalytic site. Similarly, in fructose-1,6-bisphosphatase (FBP1/PDB: 4MJO), residues N212 and D323 displayed marked entropy changes in all six pathogenic variants.

It is important to reiterate that the benign variants analyzed across all eight proteins showed negligible entropic or network deviations relative to wild-type structures. This highlights that the identified dynamic perturbations are not arbitrary consequences of amino acid substitution but are specific signatures of pathogenicity. Pathogenic variants alter the local free energy landscape, increasing conformational entropy at functional microenvironments and undermining the structural rigidity necessary for optimal functionality. Mapping recurrent network nodes and entropy-perturbed residues onto tertiary structures allowed us to delineate localized, multi-residue “candidate shared structural-dynamic perturbation patches” for each functional subclass.

It is interesting to mention that these “candidate shared structural-dynamic perturbation patches” were found to be close to either catalytic or active sites. Thus, for GALNS (4FDI), the unified patch K310–L78–N76–N289 directly encompasses the active site, where N289 coordinates the catalytic Ca^2+^ ion and Cys79 serves as the catalytic nucleophile. Similarly, for DPYS (2VR2), patch B (H248–I273) borders D326 (catalytic base and zinc ligand), while patch A (H217–R221) lies near N347 (substrate binding). Continuing with TP53RK (7SZC), the identified patches at R80 and F207 sit within critical kinase structural elements—R80 lies near E84 (which forms the conserved active-state salt bridge with K60), and F207 forms part of the hydrophobic catalytic spine (C-spine). For CRADD (2OF5), the patch L127–V160 maps directly onto the F164-centered hydrophobic core required for death domain stability and oligomerization during apoptotic signaling. Similarly, for SHMT2 (8GKT), the Y228–N379–L381 patch converges near a known neurodevelopmental disease hotspot; N379 anchors an active-site interaction network required for proper pyridoxal phosphate (PLP) positioning. Lastly, in the case of NECTIN4 (4FRW), the identified patches (R200–A202, I236, and Q163–M216–R214) map to the membrane-proximal Ig-like domains rather than the canonical D1 adhesive interface. This suggests that pathogenic mutations in NECTIN4 exert their effects via domain–domain destabilization or membrane-anchoring defects rather than direct disruption of ligand binding.

The identification of unified candidate patches across diverse mutational spectra has major implications for translational medicine and orphan drug development. As mentioned above, the standard orthosteric competitive inhibitors are often unsuitable for monogenic loss-of-function diseases because further inhibiting a compromised enzyme exacerbates the condition. The candidate patches identified here represent candidate functional patches. Binding a small molecule, pharmacological chaperone, or synthetic binder at these sites could lower side-chain entropy back to wild-type levels and restore functional long-range communication.

Identification of such common patches could help overcome the rare disease feasibility barrier, since clinical trial recruitment for rare monogenic diseases is chronically bottlenecked by small patient populations, further fragmented by genotype-specific trial designs. Demonstrating that multiple distinct mutations converge on a single molecular mechanism allows for genotype-agnostic patient stratification. A single drug targeting a shared candidate patch could treat patients carrying dozens of different pathogenic variants, drastically expanding clinical trial eligibility pools and commercial viability.

While this study offers a compelling biophysical paradigm, several limitations should be noted. Although an aggregate simulation time of 3 μs per system (3 replicas ×1 μs) captures microsecond dynamics, conformational shifts occurring on the millisecond-to-second timescale (such as large-scale domain motions or complete unfolding transitions) may require enhanced sampling methodologies like metadynamics or accelerated MD. Furthermore, some investigated targets function as dimers or higher-order oligomers in vivo (e.g., SHMT2 and DPYS). While monomeric models allowed for rigorous, isolated residue network calculations, inter-subunit network communication could reveal additional candidate patches located across protomer interfaces. Furthermore, we would like to point out that the identified residues may represent structurally sensitive positions without being causal mediators of dysfunction. Thus, the reported common candidate intervention regions may differ from putative therapeutic sites. Furthermore, it should be mentioned that no sensitivity test was done with respect to the values of various parameters and protocol details.

## 4. Methods

### 4.1. Variant Dataset Curation and Functional Subclass Annotation

A protein-focused dataset was curated from the database of monogenic disorders (MOGEDO) [34] for structure- and function-based analyses of pathogenic and benign variants. Each protein entry was annotated with the corresponding gene symbol, protein identifier, pathogenic and benign mutation information, functional subclass, and available structural information. Proteins were assigned to functional subclasses using molecular-function annotations from Gene Ontology and UniProt [44,45]. These annotations were consolidated into broader functional categories, including enzyme-related, DNA/RNA-binding, receptor, regulatory, kinase, receptor kinase, cytoskeleton/adhesion, ion channel, transporter, phosphatase, binding, and protein-folding subclasses. Ambiguous or overlapping assignments were subsequently reviewed manually to maintain consistency across the functional subclasses.

### 4.2. Identification and Selection of Representative Wild-Type Structures

Experimental structural candidates were identified for each UniProt accession using cross-references from UniProt and the Protein Data Bank (PDB) [45,46]. Candidate structures were screened to identify strict wild-type templates whenever possible, with priority given to X-ray crystallographic structures. Structures containing mutations or other non-wild-type construct modifications were excluded.

Candidate structures were evaluated for coverage of the canonical protein sequence and the annotated pathogenic and benign variant positions. For structures containing multiple chains, the chain corresponding to the target UniProt entry was selected based on UniProt-to-chain mapping and residue coverage. Protein coordinate records were retained for structural comparison, while waters, ions, ligands, cofactors, and other nonstandard structural components were removed. For proteins with multiple suitable wild-type structures, pairwise structural comparisons were performed using TM-align (version 20220412) [47]. All candidates associated with the same UniProt accession were compared in an all-versus-all manner. Because TM-align reports two TM-scores normalized independently by the lengths of the compared structures, the mean of these values was used as a symmetric TM-score. Candidate structures were ranked primarily according to their mean symmetric TM-score relative to the other available structures, while sequence coverage, mutation-position coverage, experimental method, and structural resolution were considered as additional selection criteria. When only one suitable wild-type structure was available, that structure was retained directly.

The selected per-protein representatives were subsequently grouped according to their functional subclasses. For subclasses containing multiple proteins, representative structures were again compared pairwise using TM-align, and the mean symmetric TM-score was used as the primary measure of structural representativeness. Sequence coverage, mutation-position coverage, and structural resolution were also considered. When only one eligible protein was available within a subclass, that protein was retained directly as the representative.

Eight proteins representing the other enzyme, DNA/RNA-binding, binding, receptor, regulatory, kinase, transporter, and phosphatase subclasses were ultimately selected for molecular dynamics simulations and are summarized in Table 1. Before final selection, candidate structures were additionally inspected for unresolved internal regions, noncanonical residues, structural tags, mutation-position coverage, and other features for molecular dynamics preparation. When suitable alternatives were available within the same functional subclass, structures requiring extensive reconstruction were deprioritized.

### 4.3. Residue Mapping and Structural Validation

For each selected representative, residue-level mapping was performed to establish correspondence between the experimental PDB structure and the canonical UniProt sequence. DBREF information and UniProt-to-chain mappings were used to relate PDB residue numbering to canonical UniProt numbering. The amino-acid identity of each resolved structural residue was compared with the corresponding UniProt residue, while unresolved positions were recorded separately rather than treated as sequence mismatches. Local sequence context was additionally examined where necessary to identify possible numbering offsets or sequence inconsistencies. Pathogenic and benign variant positions were then mapped onto the validated structures and classified according to whether the corresponding residue was structurally resolved, unresolved, outside the available structural coverage, or required further review. HETATM records occurring within the mapped protein sequence were inspected individually to distinguish nonstandard protein residues from crystallographic components. For example, the nonstandard residue DDZ79 in GALNS/4FDI occurred within the mapped protein region and was retained for subsequent replacement with the corresponding canonical cysteine residue. When alternate atomic conformations were present, the conformer with the highest occupancy was retained; when occupancies were equivalent, conformer A was selected. The cleaned structures were inspected using PyMOL (version 2.5.0) [48], and structural superposition was performed using the ‘align’ and ‘cealign’ commands. RMSD values, together with visual inspection, were used to identify possible chain-selection, coordinate, or truncation errors. Residue numbering in the figures and reported results follows the original PDB numbering obtained from the RCSB Protein Data Bank, rather than the local residue numbering assigned during structural preparation using pdb4amber. The original PDB residue numbering corresponds directly to UniProt numbering for all investigated proteins except 4MJO, where the UniProt residue number is one position higher than the corresponding original PDB residue number. Chain A was selected for all protein structures except 6E7K, for which chain C was used.

### 4.4. Structural Preparation and Model Generation

The final representative structures were inspected for unresolved internal regions, missing backbone atoms, incomplete side-chains, noncanonical residues, residue-numbering offsets, and structural tags before simulation. Structural repairs were performed using the previously established PDB-to-UniProt residue mapping to ensure that reconstructed residues and sequence regions corresponded to the canonical protein sequence.

Missing structural regions were reconstructed using MODELLER version 10.8 [49]. Sequence alignments between the experimental structural template and the complete target sequence were prepared using an alignment.ali file, and unresolved loop regions were modeled using the loopmodel class. When multiple candidate models were generated, structures were ranked using the Discrete Optimized Protein Energy (DOPE) score [50], and the model with the lowest DOPE score was selected for subsequent preparation and molecular dynamics simulations. Incomplete side-chains were rebuilt where necessary, and noncanonical residues within the canonical protein sequence were replaced with their corresponding UniProt residue identities. Among the final representative structures, residues 62–69 in chain A of FBP1 (4MJO), residues 298–302 in chain A of SHMT2 (8GKT), and residues 38–48 in chain A of DPYS (2VR2) were reconstructed using MODELLER. The complete DOPE score distributions are provided in Supplementary Table S6 of the accompanying Excel file. In NECTIN4 (4FRW), an additional histidine residue present in the PDB structure but absent from the corresponding reference FASTA sequence was removed using UCSF ChimeraX (version 1.12) [51], and a truncated side-chain was rebuilt using MODELLER [49]. The resulting structures were inspected for residue completeness, sequence consistency, residue numbering, and overall structural geometry. The prepared wild-type structures were subsequently used as templates for constructing the corresponding benign and pathogenic systems for molecular dynamics simulations.

### 4.5. Molecular Dynamics Simulations

Molecular dynamics (MD) simulations were carried out for the finalized representative protein systems using the GPU-enabled pmemd.CUDA engine in AMBER24 (version 24) [52]. Prior to system preparation, only the protein structures were retained by removing all other molecules present in the original structures. The resulting structures were protonated using PDB2PQR (version 3.7.1) [53], with protonation states of titratable residues assigned at physiological pH 7.0 using the PROPKA (version 3.5.1) algorithm [54]. The protonated structures were then further processed using the ‘tleap’ module in AMBER24. The protein was parameterized using the ff19SB force field [55]. Each system was solvated in a cuboid box of explicit OPC water [56], with a 14 Å buffer around the solute, and Na^+^ or Cl^−^ counterions were added as required to neutralize the system. Each solvated system was first subjected to three stages of restrained energy minimization. Each minimization consisted of 5000 steps, including 2500 steps of steepest descent (SD) followed by 2500 steps of conjugate gradient (CG). Positional restraints were applied to the protein heavy atoms and gradually decreased from 100 to 50 and 25 kcal·mol^−1^·Å^−2^ over the three minimization stages. Following minimization, the systems were heated from 0 to 300 K in six stages using the NVT ensemble, with the temperature increased by 50 K over 10 ps in each stage at a 1 fs timestep. During heating, a positional restraint of 25 kcal·mol^−1^·Å^−2^ was applied to the protein heavy atoms. Following heating, five additional restrained minimizations (1000 steps, 500 steps SD followed by 500 steps of CG) were performed, with positional restraints on the protein heavy atoms gradually decreased to 25, 10, 5, 2, and 1 kcal·mol^−1^·Å^−2^. The systems were then equilibrated at 300 K through four successive 100 ps simulations in the NVT ensemble, with restraints decreased stepwise to 10, 5, 2, and 1 kcal·mol^−1^·Å^−2^ at time step 2 fs. This was followed by four 100 ps equilibration stages in the NPT ensemble at 300 K and 1 bar using the same decreasing restraint schedule of 10, 5, 2, and 1 kcal·mol^−1^·Å^−2^ at timestep 2 fs. Following the restrained equilibration, the systems underwent additional unrestrained NPT equilibration stages of 100 ps and 1 ns at 300 K and 1 bar. The 100-ps and 1-ns unrestrained equilibration stages were excluded from downstream analyses. The resulting equilibrated systems were then used to initiate the production simulations. Production simulations were carried out without restraints in the NPT ensemble at 300 K and 1 bar using a 2 fs time step. Bonds involving hydrogen atoms were constrained using the SHAKE algorithm [57]. Temperature was maintained at 300 K using a Langevin thermostat [58] with a collision frequency of 1.0 ps^−1^. Pressure was maintained at 1 bar using an isotropic Monte Carlo barostat [59] with a pressure-relaxation time of 1 ps. Long-range electrostatic interactions were treated using the particle-mesh Ewald (PME) method [60] under periodic boundary conditions. A 10 Å cutoff was used for nonbonded interactions, with the van der Waals interactions smoothly switched between 8 and 10 Å. Energies and coordinates were saved every 20 ps. Three independent replicas were generated for each system, with the replicas initiated using different random seeds for velocity assignment. Each replica consisted of 1 μs of unrestrained production simulation, resulting in a total production simulation time of 3 μs per system. As part of the simulation quality-control analysis, protein RMSD profiles were examined independently for each production replica and are provided in Supplementary Figures S1–S8. Following the simulations, trajectory post-processing and subsequent structural analyses were performed using ‘cpptraj’ (version 6.24.0) [61]. For post-processing, each individual trajectory was re-imaged to correct for periodic boundary conditions, and stripped of solvent and ions as appropriate, and then these trajectories were concatenated for the subsequent network and entropy analysis.

### 4.6. Dynamic Network Analysis

Residue-based dynamic network analysis was performed using the Python package DyNetAn (version 2.3.0) [62], following the general workflow described in the original method paper [63]. For each WT, benign, and pathogenic system, the three independent 1-μs production trajectories were concatenated to generate a combined 3-μs analysis trajectory. Solvent molecules were excluded before network construction, and the protein structures were structurally aligned prior to analysis. The entire concatenated 3-μs trajectory was analyzed as a single analysis window.

The simulated frames were represented as residue interaction networks, with each protein residue modeled as a node defined by its Cα atom. An edge was established between two nodes when the minimum distance between any pair of heavy atoms belonging to the respective residues remained within 4.5 Å for at least 75% of the analyzed frames. Contact searching was performed using every fifth trajectory frame. Contacts involving atoms within the same residue and contacts between consecutive residues were excluded to reduce trivial local backbone connectivity, and isolated nodes were removed before subsequent network calculations. Generalized correlations between the motions of connected nodes were calculated using DyNetAn and used to construct the correlation-weighted network. Optimal communication pathways were subsequently determined, and edge betweenness centrality, eigenvector centrality, and network communities were calculated. For each residue, a node-level bottleneck score was calculated as the sum of the edge-betweenness centrality values of all edges incident on that node. Residues were ranked according to their bottleneck scores, and the 25 highest-ranking residues from each system were retained for subsequent comparative analysis. To identify bottleneck positions conserved under a nonpathogenic perturbation, the top-25 bottleneck residues from the WT system were first compared with those identified in the corresponding benign variant. A WT residue was retained as part of the reference network when the same residue position was also present among the top-25 bottleneck residues of the benign system. When an identical residue position was not identified, spatial correspondence was evaluated using the average structure obtained from the benign MD trajectory. In this case, a WT bottleneck residue was retained in the reference set when at least one of the benign top-25 bottleneck residues were located within 5.0 Å based on the Cα–Cα distance. Thus, the resulting WT/benign reference network represented WT bottleneck positions that were preserved in the benign system either at the identical residue position or at a spatially corresponding position. WT bottleneck residues without either direct or spatial correspondence in the benign system were excluded from the reference set.

Each pathogenic variant was then compared independently with this WT/benign reference signature network. A reference residue was classified as shared-direct when the identical residue position was present among the top-25 bottleneck residues of the pathogenic variant. When the identical position was absent, spatial correspondence was evaluated using the average structure obtained from the corresponding pathogenic trajectory. A reference residue was classified as shared-spatial when at least one of the pathogenic top-25 bottleneck residues was located within 5.0 Å of the reference residue based on the Cα–Cα distance. A reference-network residue for which neither direct nor spatial correspondence was identified among the pathogenic top-25 bottleneck residues was classified as a Network Loss. Conversely, a pathogenic top-25 bottleneck residue that could not be associated with any residue in the WT/benign reference network by either direct or spatial matching was classified as a Network Gain. Gain and loss events were subsequently compared across all pathogenic variants. For each residue, recurrence was defined as the number of pathogenic variants exhibiting the corresponding gain or loss event out of the total number of pathogenic variants analyzed.

### 4.7. Side-Chain Entropy

Side-chain conformational entropy was analyzed from the MD trajectories to characterize residue-level changes in side-chain conformational sampling. For each WT, benign, and pathogenic system, the *χ*_1_ and *χ*_2_ side-chain dihedral angles were extracted for all applicable protein residues. All saved trajectory frames were included, corresponding to the complete 1 μs production trajectory from each of the three independent replicas. For residues containing both *χ*_1_ and *χ*_2_ dihedral angles, each torsion was divided into three rotameric states, and the two dihedral angles were combined to generate a 3 × 3, or nine-state, joint *χ*_1_/*χ*_2_ representation. The symmetry of the *χ*_2_ torsion for Asp, Phe, and Tyr residues was accounted for using a 180° periodicity. For each system, the three independent replica trajectories were pooled, and the probability (*p_i_*) of occupying each of the nine joint rotameric states was calculated.

Side-chain conformational entropy was calculated using the Shannon entropy expression [64],

$$S=-\sum_{i}\;p_{i\;}\ln p_{i}$$

and normalized by ln(9), corresponding to the maximum possible entropy for a nine-state joint distribution. The normalized entropy ranges from 0 to 1. A value approaching 0 indicates that a residue predominantly occupies a single *χ*_1_/*χ*_2_ rotameric state, corresponding to relatively restricted side-chain conformational sampling. In contrast, a value approaching 1 indicates increasingly broad sampling across the nine possible joint rotameric states, with the maximum value corresponding to equal occupancy of all nine states.

For each residue, the normalized entropy of the benign and pathogenic systems was compared with the corresponding WT value:

*ΔS* = *S_variant_* − *S*_WT_

Accordingly, positive *ΔS* values indicate increased side-chain conformational diversity relative to WT, whereas negative values indicate reduced conformational sampling or increased restriction of the side-chain. Because both the variant and WT normalized entropies are bounded between 0 and 1, the theoretical range of *ΔS* is −1 to +1. A residue was considered pathogenically perturbed when the magnitude of its normalized entropy change exceeded 0.10 in a pathogenic variant, while the corresponding entropy change in the benign variant remained below 0.05. These thresholds were used as operational criteria to distinguish comparatively large pathogenic alterations in side-chain conformational sampling from residues whose conformational behavior remained close to the WT state in the benign system. Residues that satisfied these criteria across multiple pathogenic variants were identified as recurrent side-chain entropy perturbations.

## 5. Conclusions

In summary, this study demonstrates that despite extensive mutational diversity in monogenic disorders, most pathogenic variants converge on shared, localized structural and dynamic perturbations. By integrating molecular dynamics simulations, dynamic network centralities, and side-chain entropic metrics across eight representative protein families, we identified candidate shared structural-dynamic perturbation patches that account for disease mechanisms across multiple genotypes. This shift from a “one-mutation, one-drug” paradigm toward a mechanism-based, candidate functional patch-targeting framework provides a foundation for designing broad-spectrum therapeutics capable of rescuing protein function across heterogeneous patient populations.

## Figures and Tables

**Table 1:** Final protein targets selected for downstream molecular dynamics and residue-network analyses.

Functional Subclass	Protein	UniProt ID	Representative PDB
Other enzyme	N-acetylgalactosamine-6-sulfatase (GALNS)	P34059	4FDI
DNA/RNA-binding	Serine hydroxymethyltransferase, mitochondrial (SHMT2)	P34897	8GKT
Binding	Dihydropyrimidinase (DPYS)	Q14117	2VR2
Receptor	Nectin-4 (NECTIN4)	Q96NY8	4FRW
Regulatory	Death domain-containing protein CRADD (CRADD)	P78560	2OF5
Kinase	EKC/KEOPS complex subunit TP53RK (TP53RK)	Q96S44	7SZC
Transporter	Glycosylphosphatidylinositol-anchored high-density lipoprotein-binding protein 1 (GPIHBP1)	Q8IV16	6E7K
Phosphatase	Fructose-1,6-bisphosphatase 1 (FBP1)	P09467	4MJO

**Table 2:** Recurrent Network Gain and Loss residues identified across the eight representative proteins. Values in parentheses indicate recurrence, expressed as the number of pathogenic variants in which a given network alteration was observed divided by the total number of pathogenic variants analyzed for that protein. For example, 3/3 indicates that the alteration was observed in all three pathogenic variants, whereas 2/3 indicates that it was observed in two of the three pathogenic variants.

Protein (PDB ID)	Recurrent Network Gain	Recurrent Network Loss
Death domain-containing protein CRADD (CRADD) (2OF5)	I145 (3/3); L127 (2/3); V160 (2/3)	
Dihydropyrimidinase (DPYS) (2VR2)	H248 (5/6); I273 (4/6)	H217 (4/6)
N-acetylgalactosamine-6-sulfatase (GALNS) (4FDI)	K310 (6/6); H236 (5/6); Y385 (5/6); L78 (4/6); E320 (4/6)	N76 (5/6)
Nectin-4 (NECTIN4) (4FRW)	R200 (3/3); A202 (3/3); I236 (3/3); R69 (2/3); S176 (2/3)	
Fructose-1,6-bisphosphatase 1 (FBP1) (4MJO)	Y226 (6/6); L166 (4/6)	V249 (5/6); L34 (4/6)
Glycosylphosphatidylinositol-anchored high-density lipoprotein-binding protein 1 (GPIHBP1) (6E7K)	T90 (5/6); T124 (3/6)	L63 (3/6)
EKC/KEOPS complex subunit TP53RK (TP53RK) (7SZC)	R80 (3/3); F207 (3/3); C110 (2/3); S187 (2/3)	F32 (2/3); F221 (2/3)
Serine hydroxymethyltransferase, mitochondrial (SHMT2) (8GKT)	Y228 (3/4); L258 (3/4); T275 (3/4); N379 (3/4); L381 (3/4)	

**Table 3:** Recurrent side-chain entropy perturbations were identified across the eight investigated proteins. Values in parentheses indicate recurrence, expressed as the number of pathogenic variants in which a residue showed an entropy perturbation divided by the total number of pathogenic variants analyzed for that protein. For example, 6/6 indicates perturbation in all six pathogenic variants, whereas 4/6 indicates perturbation in four of the six variants.

Protein (PDB ID)	Recurrently Perturbed Side-Chain Entropy Residues
Death domain-containing protein CRADD (CRADD) (2OF5)	L194 (2/3)
Dihydropyrimidinase (DPYS) (2VR2)	W120 (6/6); Q71 (5/6); H69 (5/6); R221 (5/6); N347 (4/6)
N-acetylgalactosamine-6-sulfatase (GALNS) (4FDI)	R253 (5/6); R105 (5/6); Y108 (5/6); H398 (5/6); N289 (4/6); L395 (4/6); F167 (4/6)
Nectin-4 (NECTIN4) (4FRW)	M216 (3/3); Q163 (3/3); R214 (3/3); R97 (2/3)
Fructose-1,6-bisphosphatase 1 (FBP1) (4MJO)	N212 (6/6); D323 (6/6); F117 (5/6); K230 (5/6); P233 (4/6); D251 (4/6)
Glycosylphosphatidylinositol-anchored high-density lipoprotein-binding protein 1 (GPIHBP1) (6E7K)	L63 (2/6); R64 (2/6); E122 (2/6)
EKC/KEOPS complex subunit TP53RK (TP53RK) (7SZC)	N109 (2/3); Y106 (2/3)
Serine hydroxymethyltransferase, mitochondrial (SHMT2) (8GKT)	Y106 (3/4); H171 (3/4); Y141 (2/4); M498 (2/4); Q324 (2/4)

## Data Availability

The data supporting the findings of this study are included in the manuscript and the accompanying Supplementary Materials. Additional data are available from the corresponding author upon reasonable request.

## Supplementary Materials

The following supporting information can be downloaded at https://www.mdpi.com/article/10.3390/ijms27219432/s1.
