# Supplementary material for: Beyond Mutation Diversity: Common Pathogenic Mechanism for Putative Intervention Sites in Monogenic Diseases
Source: Int J Mol Sci. 2026 Oct 22;27(21):9432. doi: 10.3390/ijms27219432 (PMC13763534; doi:10.3390/ijms27219432)
Supplement: Supplementary file 1 [file ijms-27-09432-s001.zip › ijms-4626141 supplementary figures.pdf]

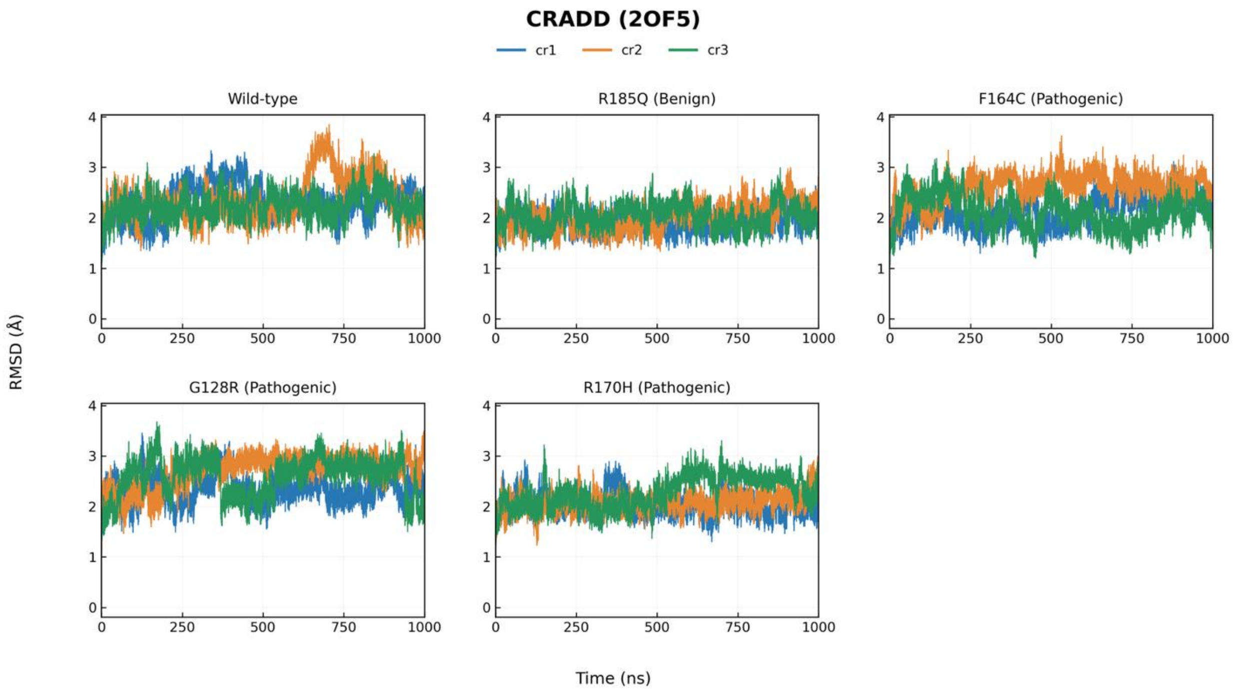

Supplementary Figure S1. Root-mean-square deviation (RMSD) profiles of CRADD (PDB ID: 2OF5) and its variants. RMSD profiles from three independent 1-μs molecular dynamics simulations of the wild-type (WT), benign variant R185Q, and pathogenic variants F164C, G128R, and R170H. The three replicas (cr1, cr2, and cr3) are represented by blue, orange, and green curves, respectively. RMSD values are expressed in angstroms (Å) as a function of simulation time (ns).

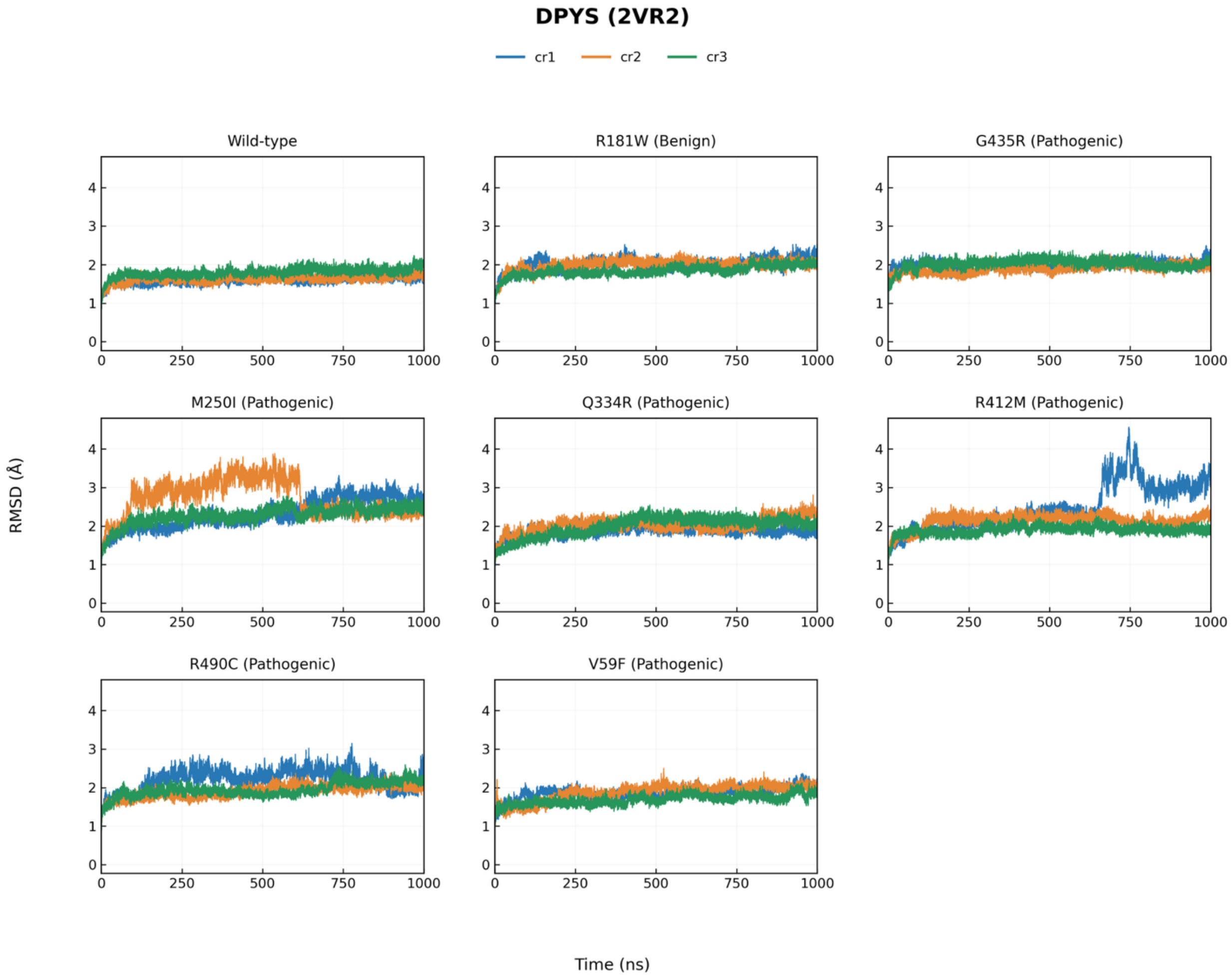

Supplementary Figure S2. Root-mean-square deviation (RMSD) profiles of DPYS (PDB ID: 2VR2) and its variants. RMSD profiles from three independent 1-μs molecular dynamics simulations of the wild-type (WT), benign variant R181W, and pathogenic variants G435R, M250I, Q334R, R412M, R490C, and V59F. The three replicas (cr1, cr2, and cr3) are represented by blue, orange, and green curves, respectively. RMSD values are expressed in angstroms (Å) as a function of simulation time (ns).

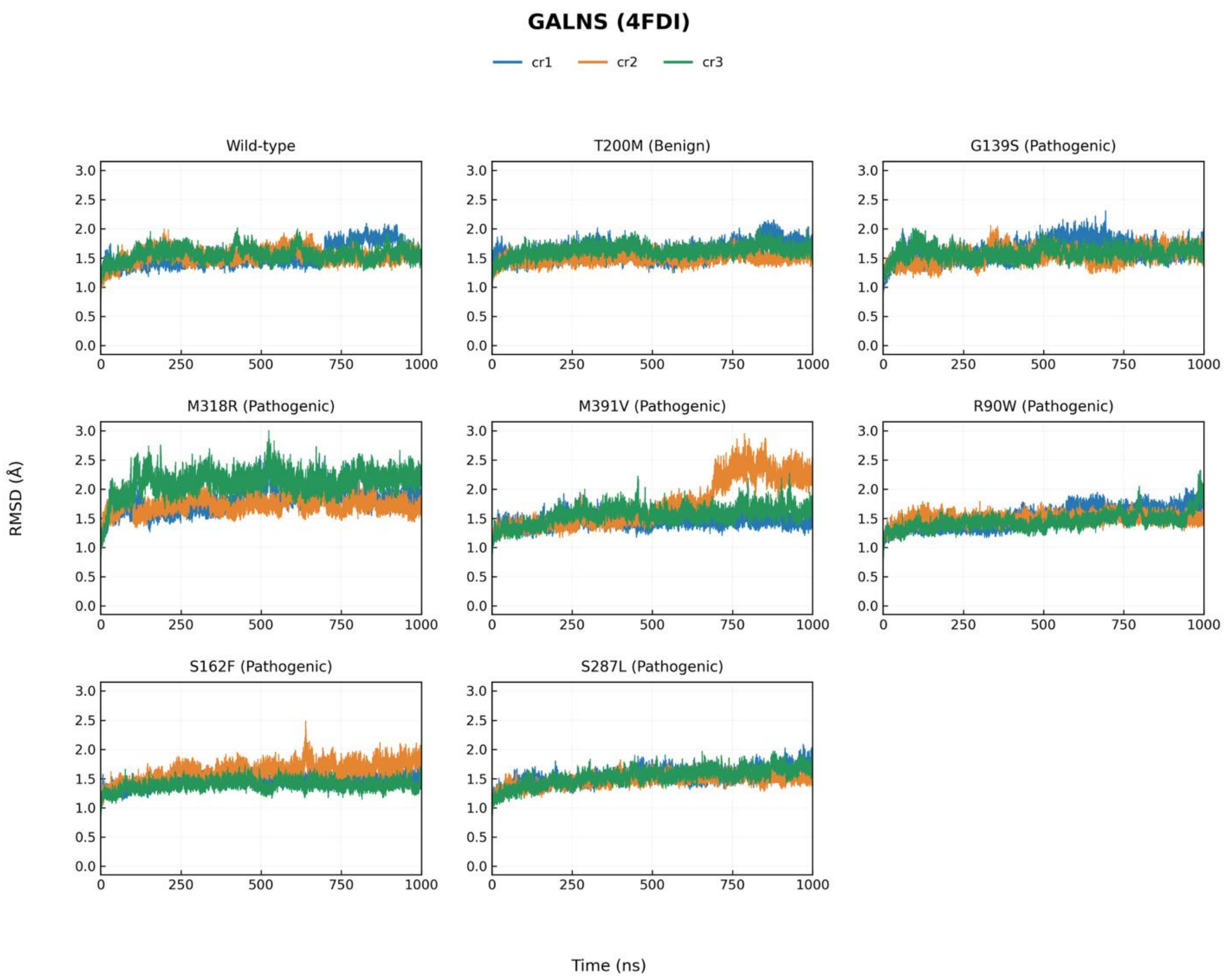

Supplementary Figure S3. Root-mean-square deviation (RMSD) profiles of GALNS (PDB ID: 4FDI) and its variants. RMSD profiles from three independent 1-µs molecular dynamics simulations of the wild-type (WT), benign variant T200M, and pathogenic variants G139S, M318R, M391V, R90W, S162F, and S287L. The three replicas (cr1, cr2, and cr3) are represented by blue, orange, and green curves, respectively. RMSD values are expressed in angstroms (Å) as a function of simulation time (ns).

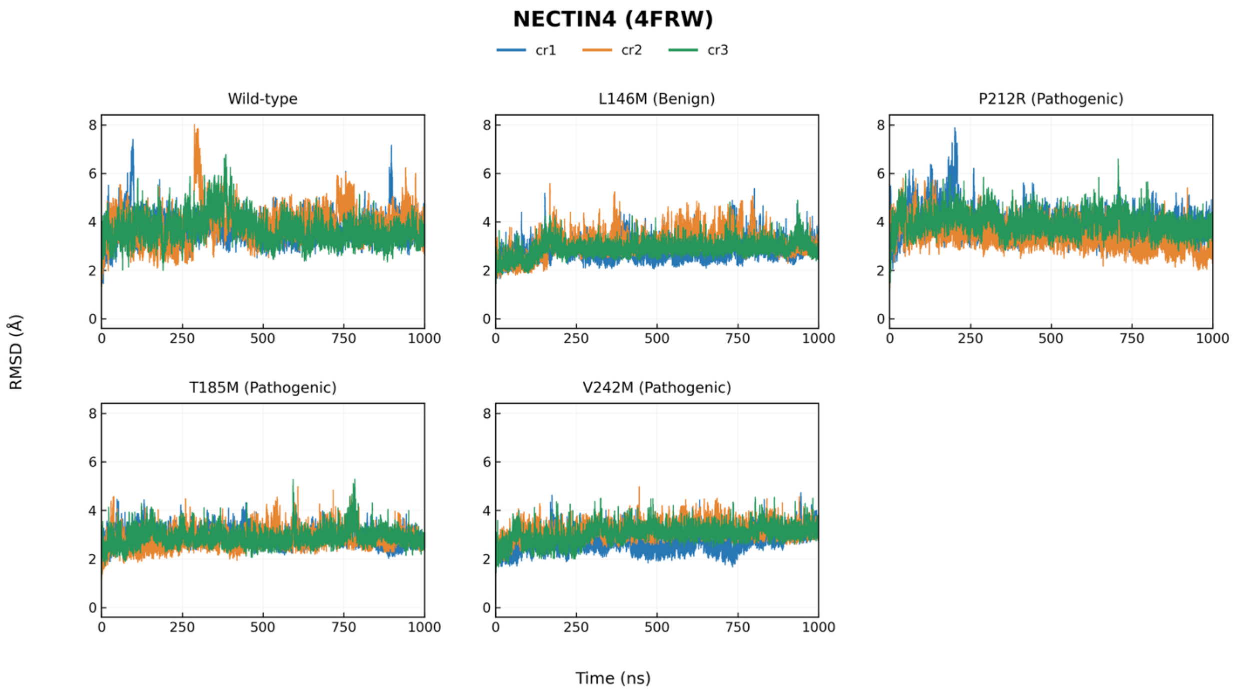

Supplementary Figure S4. Root-mean-square deviation (RMSD) profiles of NECTIN4 (PDB ID: 4FRW) and its variants. RMSD profiles from three independent 1-µs molecular dynamics simulations of the wild-type (WT), benign variant L146M, and pathogenic variants P212R, T185M, and V242M. The three replicas (cr1, cr2, and cr3) are represented by blue, orange, and green curves, respectively. RMSD values are expressed in angstroms (Å) as a function of simulation time (ns).

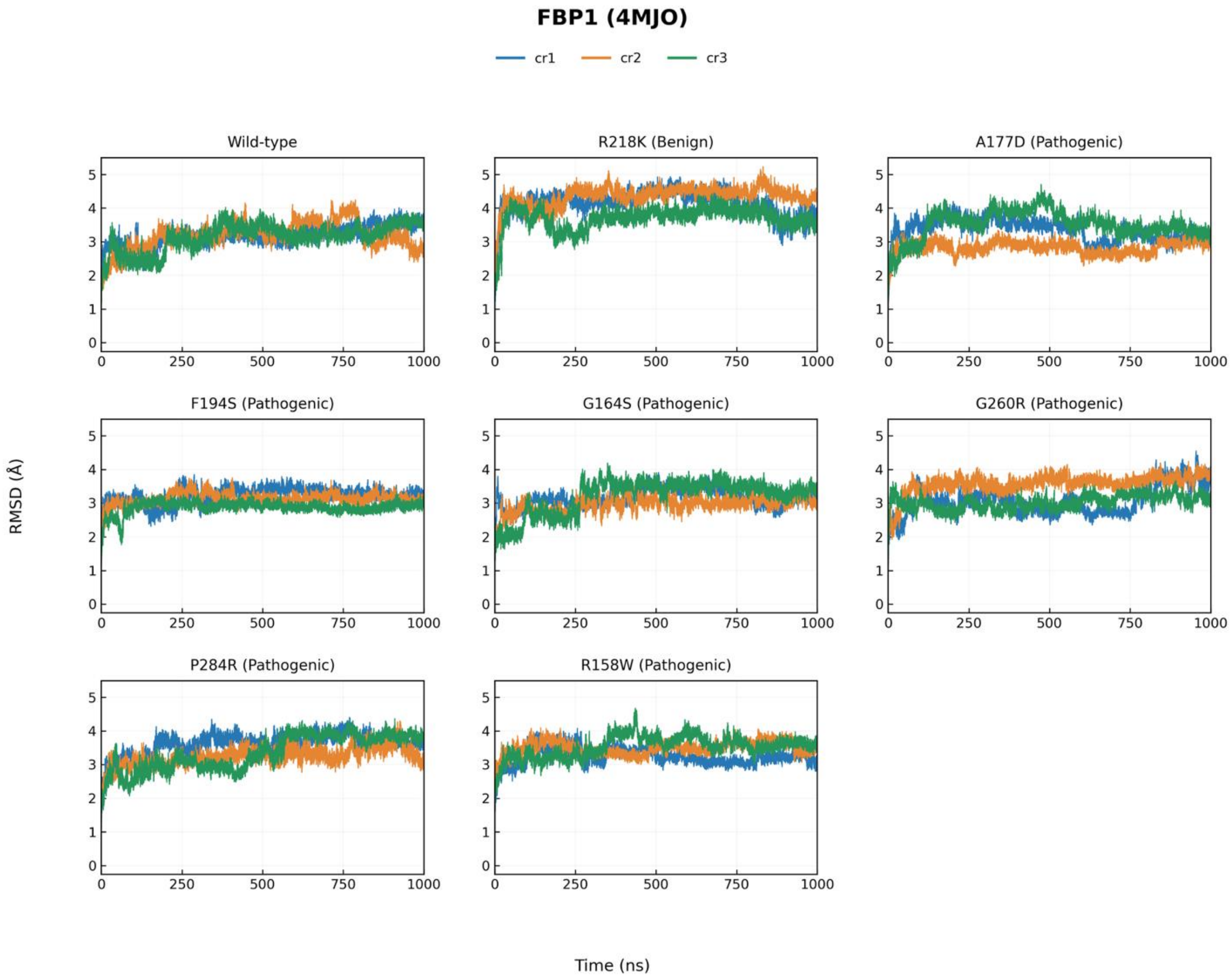

Supplementary Figure S5. Root-mean-square deviation (RMSD) profiles of FBP1 (PDB ID: 4MJO) and its variants. RMSD profiles from three independent 1-μs molecular dynamics simulations of the wild-type (WT), benign variant R218K, and pathogenic variants A177D, F194S, G164S, G260R, P284R, and R158W. The three replicas (cr1, cr2, and cr3) are represented by blue, orange, and green curves, respectively. RMSD values are expressed in angstroms (Å) as a function of simulation time (ns).

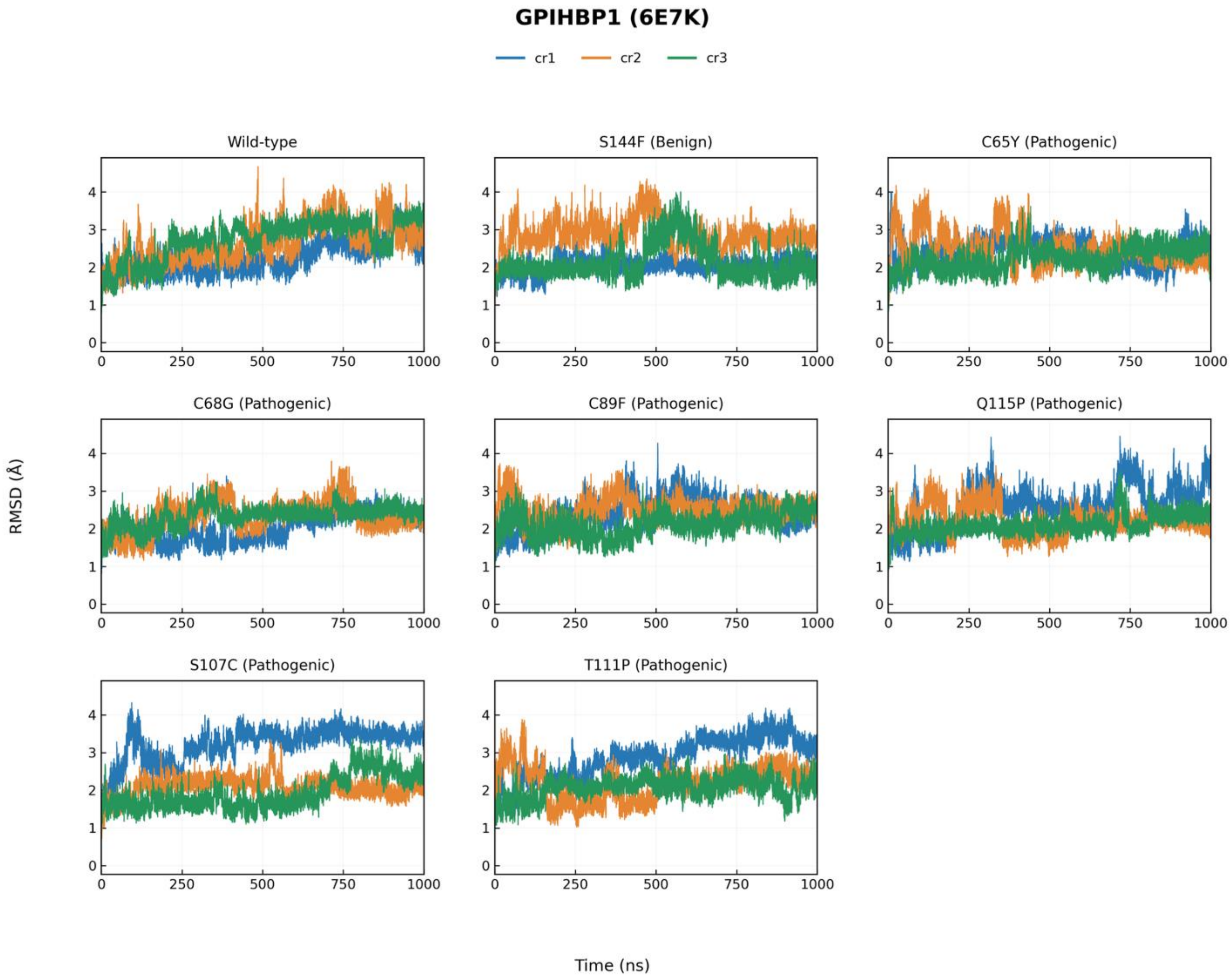

Supplementary Figure S6. Root-mean-square deviation (RMSD) profiles of GPIHBP1 (PDB ID: 6E7K) and its variants. RMSD profiles from three independent 1-µs molecular dynamics simulations of the wild-type (WT), benign variant S144F, and pathogenic variants C65Y, C68G, C89F, Q115P, S107C, and T111P. The three replicas (cr1, cr2, and cr3) are represented by blue, orange, and green curves, respectively. RMSD values are expressed in angstroms (Å) as a function of simulation time (ns).

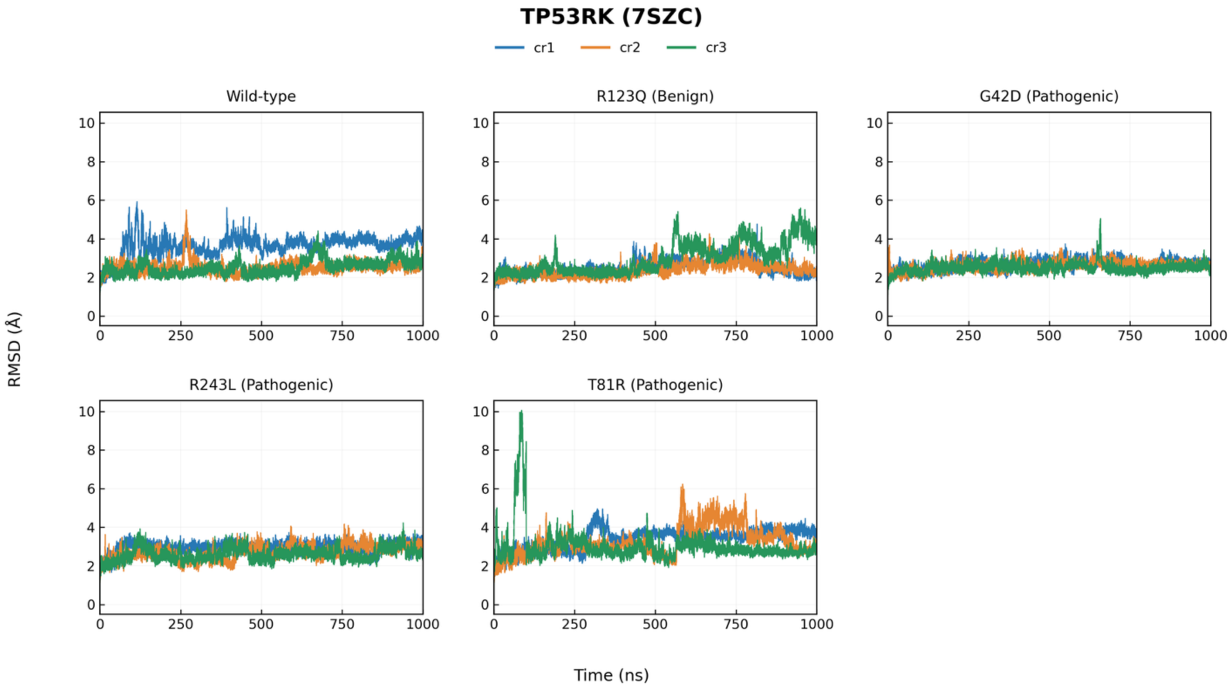

Supplementary Figure S7. Root-mean-square deviation (RMSD) profiles of TP53RK (PDB ID: 7SZC) and its variants. RMSD profiles from three independent 1-µs molecular dynamics simulations of the wild-type (WT), benign variant R123Q, and pathogenic variants G42D, R243L, and T81R. The three replicas (cr1, cr2, and cr3) are represented by blue, orange, and green curves, respectively. RMSD values are expressed in angstroms (Å) as a function of simulation time (ns).

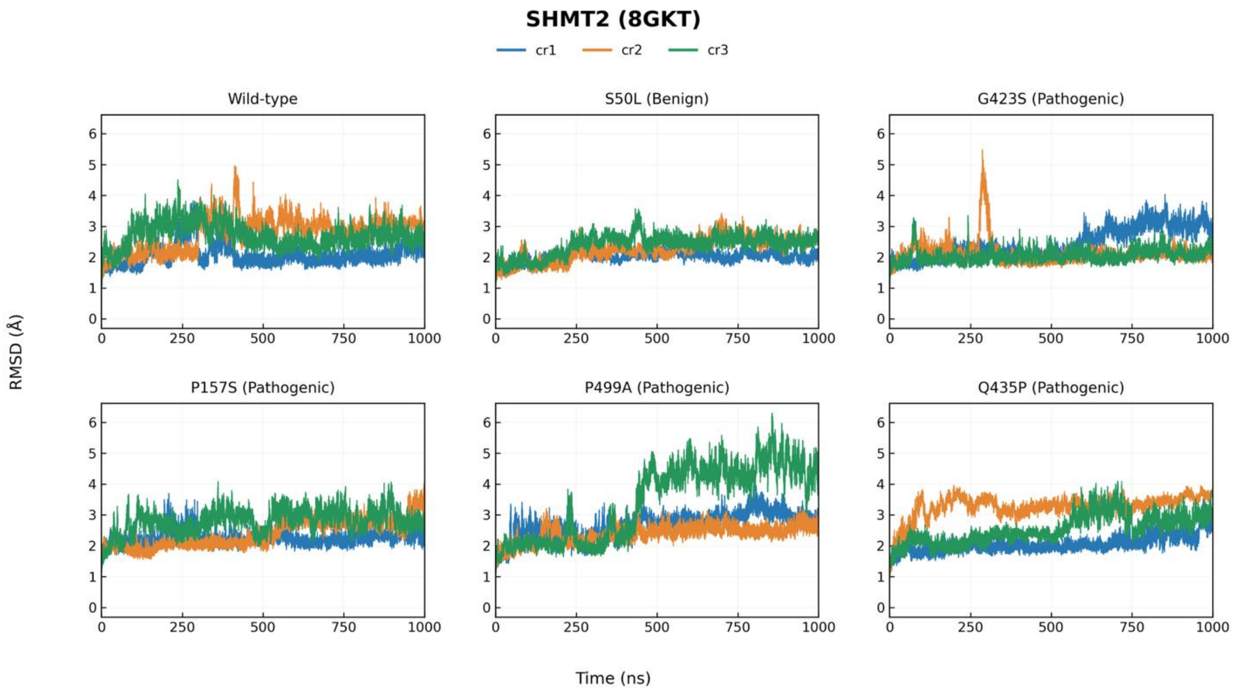

Supplementary Figure S8. Root-mean-square deviation (RMSD) profiles of SHMT2 (PDB ID: 8GKT) and its variants. RMSD profiles from three independent 1-μs molecular dynamics simulations of the wild-type (WT), benign variant S50L, and pathogenic variants G423S, P157S, P499A, and Q435P. The three replicas (cr1, cr2, and cr3) are represented by blue, orange, and green curves, respectively. RMSD values are expressed in angstroms (Å) as a function of simulation time (ns).
